# Spatiotemporal dynamics in spiking simulations of superior colliculus fit via MCMC suggest disinhibition responsible for superlinear summation

**DOI:** 10.1186/1471-2202-16-S1-P293

**Published:** 2015-12-18

**Authors:** Richard Veale, Tadashi Isa, Masatoshi Yoshida

**Affiliations:** 1 National Institute for Physiological Sciences, Okazaki, Japan; 2 SOKENDAI (The Graduate University for Advanced Studies), Hayama, Japan

## 

**Figure 1:**
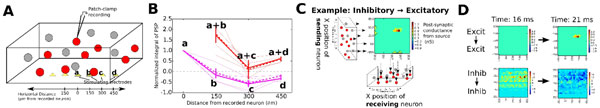
**(A) Experimental setup of **[1], **showing slice containing excitatory (red) and inhibitory (gray) neurons**. Stimulation applied to electrodes at lateral distances while recording from an excitatory neuron. **(B) **Empirical results (dotted) and model fits. Purple: repetitive stimulation single point, Red: repetitive stimulation two point. **(C) **Example of a dynamics plot, in this case Inhib→Excit, showing net conductance in circuit as a function of sending and receiving neuron. **(D) ***Difference *between dynamics plots. Two-point stimulation minus the linear sum of two single-point stimulation conditions, showing where and when non-linear changes in activity occur.

The superior colliculus (SC) is a midbrain region with visually responsive neurons in the superficial layers and eye movement controlling neurons in the deeper layers. Recently, [1] performed *in vitro *experiments to elucidate lateral interactions within horizontal slices of the SC (Figure 1A). The experiments indicate that the superficial (visual) layers implement surround inhibition, and furthermore that strong stimulation at two adjacent locations (separation ~150 μm) produces an unexpected super-linear summation that is not seen in the deeper layers (Figure 1B, a**+b**). We used differential evolution Markov-chain Monte Carlo (MCMC) to estimate the parameters of a large-scale spiking neural circuit simulation to fit the slice data [2]. The model contains populations of inhibitory and excitatory neurons as well as input axons from retina/cortex. We included free anatomical (dendrite/axon spread) and dynamic (short-term synaptic plasticity) hyper-parameters in the model, and used MCMC estimate the posterior distribution of parameters that is most likely given the slice data. The resulting marginal distributions show promising agreement with verifiable anatomical parameters such as the lateral spread of dendrites and axons of the inhibitory and excitatory neuron populations in the superficial colliculus, even though no such constraints were coded into the model [2]. However, the posterior distributions for non-intuitive parameters (such as synaptic efficacies and facilitation/depression time constants) cannot be verified directly with existing data. Furthermore, it is not clear what the role of the dynamical parameters is in producing the behavior of the best-fit models, for example the local superlinearity described above. In this work, we take the additional step of analyzing the spatio-temporal dynamics of one of the best-fit regions of parameter space found via MCMC. The purpose is to provide a mechanistic explanation for the super-linear summation observed during two-point stimulation. Figure 1D shows the difference in spatio-temporal dynamics between actual two-point stimulation versus the linear sum of two independent stimulations. Thus, positive values indicate an increase in conductance sent to neurons at the horizontal axis position, arriving from neurons at the position indicated by the vertical axis (as explained in 1C). 16 ms from stimulation onset, there is a large increase in flow of inhibitory input from near the center of the circuit to inhibitory neurons all around the circuit, thus *disinhibiting *the circuit. 5 ms later, the excitatory neurons near the center receive input from excitatory neurons near the middle (i.e. recurrent activity), suggesting that cause for the super-linearity.
